# Prediction of Long-Term Outcomes in ST-Elevation Myocardial Infarction and Non-ST Elevation Myocardial Infarction with and without Creatinine Kinase Elevation—Post-Hoc Analysis of the J-MINUET Study

**DOI:** 10.3390/jcm9082667

**Published:** 2020-08-18

**Authors:** Shigeru Toyoda, Masashi Sakuma, Shichiro Abe, Teruo Inoue, Koichi Nakao, Yukio Ozaki, Kazuo Kimura, Junya Ako, Teruo Noguchi, Satoru Suwa, Kazuteru Fujimoto, Yasuharu Nakama, Takashi Morita, Wataru Shimizu, Yoshihiko Saito, Atsushi Hirohata, Yasuhiro Morita, Atsunori Okamura, Toshiaki Mano, Minoru Wake, Kengo Tanabe, Yoshisato Shibata, Mafumi Owa, Kenichi Tsujita, Hiroshi Funayama, Nobuaki Kokubu, Ken Kozuma, Tetsuya Toubaru, Keijirou Saku, Shigeru Ohshima, Yoshihiro Miyamoto, Hisao Ogawa, Masaharu Ishihara

**Affiliations:** 1 Department of Cardiovascular Medicine, Dokkyo Medical University, Mibu 321-0293, Japan; masakuma@dokkyomed.ac.jp (M.S.); abenana@dokkyomed.ac.jp (S.A.); inouet@dokkyomed.ac.jp (T.I.); 2 Division of Cardiology, Saiseikai Kumamoto Hospital Cardiovascular Center, Kumamoto 861-4193, Japan; koichi-nakao@saiseikaikumamoto.jp; 3 Department of Cardiology, Fujita Health University, Toyoake 470-1101, Japan; ozakiyuk@fujita-hu.ac.jp; 4 Cardiovascular Center, Yokohama City University Medical Center, Yokohama 236-0004, Japan; c_kimura@yokohama-cu.ac.jp; 5 Department of Cardiovascular Medicine, Kitasato University School of Medicine, Sagamihara 252-0375, Japan; jako@jichi.ac.jp; 6 Department of Cardiovascular Medicine, National Cerebral and Cardiovascular Center, Suita 565-8565, Japan; tnoguchi@ncvc.go.jp (T.N.); ogawah@ncvc.go.jp (H.O.); 7 Department of Cardiology, Juntendo University Shizuoka Hospital, Izunokuni 410-2295, Japan; suwatetsu3000@yahoo.co.jp; 8 Department of Cardiology, National Hospital Organization Kumamoto Medical Center, Kumamoto 860-0088, Japan; fujimoto.kazuteru.yf@mail.hosp.go.jp; 9 Department of Cardiology, Hiroshima City Hospital, Hiroshima 730-8518, Japan; modified-bruce@tenor.ocn.ne.jp; 10 Division of Cardiology, Osaka General Medical Center, Osaka 558-8558, Japan; tmorita@sc4.so-net.ne.jp; 11 Department of Cardiovascular Medicine, Nippon Medical School, Tokyo 113-8603, Japan; wshimizu@nms.ac.jp; 12 First Department of Internal Medicine, Nara Medical University, Kashihara 634-8521, Japan; yssaito@naramed-u.ac.jp; 13 Department of Cardiovascular Medicine, The Sakakibara Heart Institute of Okayama, Okayama 700-0804, Japan; hirohata@tg7.so-net.ne.jp; 14 Department of Cardiology, Ogaki Municipal Hospital, Ogaki 503-8502, Japan; ysmorita@med.nagoya-u.ac.jp; 15 Department of Cardiology, Sakurabashi Watanabe Hospital, Osaka 530-0001, Japan; a_okamura@watanabe-hsp.or.jp; 16 Institute for Clinical Research, Kansai Rosai Hospital, Osaka 660-8511, Japan; mano-toshiaki@kansaih.johas.go.jp; 17 Department of Cardiology, Okinawa Chubu Hospital, Uruma 904-2293, Japan; wake_minoru@hosp.pref.okinawa.jp; 18 Division of Cardiology, Mitsui Memorial Hospital, Tokyo 101-8043, Japan; kengo-t@zd5.so-net.ne.jp; 19 Department of Cardiology, Miyazaki Medical Association Hospital, Miyazaki 880-0834, Japan; yshibata@cure.or.jp; 20 Department of Cardiovascular Medicine, Suwa Red Cross Hospital, Suwa 392-8510, Japan; mafumi-oowa@suwa.jrc.or.jp; 21 Department of Cardiovascular Medicine, Graduate School of Medical Sciences, Kumamoto University, Kumamoto 860-8556, Japan; tsujita@kumamoto-u.ac.jp; 22 Division of Cardiovascular Medicine, Department of Medicine, Jichi Medical University School of Medicine, Tochigi 329-0498, Japan; funahiro@omiya.jichi.ac.jp; 23 Department of Cardiovascular, Renal and Metabolic Medicine, Sapporo Medical University, Sapporo 060-8543, Japan; kokubu@sapmed.ac.jp; 24 Department of Cardiology, Teikyo University, Tokyo 173-8606, Japan; PXE00364@nifty.com; 25 Department of Cardiology, Sakakibara Heart Institute, Tokyo 183-0003, Japan; ttobaru9@gmail.com; 26 Department of Cardiology, Fukuoka University School of Medicine, Fukuoka 814-0133, Japan; saku-k@fukuoka-u.ac.jp; 27 Department of Cardiology, Gunma Prefectural Cardiovascular Center, Maebashi 371-0004, Japan; ohshima.s@cvc.pref.gunma.jp; 28 Department of Preventive Cardiology, National Cerebral and Cardiovascular Center, Suita 565-8565, Japan; knishimu@ncvc.go.jp; 29 Department of Cardiovascular and Renal Medicine, Hyogo College of Medicine, Nishinomiya 663-8501, Japan; ishifami@fb3.so-net.ne.jp

**Keywords:** myocardial infarction, cardiac troponin, creatine kinase, predictor of prognosis, risk score

## Abstract

Background: A Japanese prospective, nation-wide, multicenter registry (J-MINUET) showed that long-term outcomes were worse in non-ST elevation acute myocardial infarction (NSTEMI), diagnosed by increased cardiac troponin levels, compared to STEMI. This was observed in both non-STEMI with elevated creatine kinase (CK) (NSTEMI+CK) and non-STEMI without elevated CK (NSTEMI-CK). However, predictive factors for long-term outcomes in STEMI, NSTEMI+CK, and NSTEMI-CK have not been elucidated. Methods: Using the Cox proportional hazards model, we determined significant independent predictors of long-term outcomes from a total of 111 parameters evaluated in the J-MINUET study in each of our groups, including STEMI, NSTEMI+CK, and NSTEMI-CK. Then, we calculated the risk score using the regression coefficients for the determined independent predictors for the strict prediction of long-term outcomes. Results: Prognostic factors, as well as composite cardiovascular events and all-cause death, were different between STEMI, NSTEMI+CK, and NSTEMI-CK. Risk scores could effectively and powerfully predict both composite cardiovascular events and all-cause death in each group. Conclusions: The prediction of long-term outcomes using cored parameters of baseline demographics and clinical characteristics is feasible and could prove useful in establishing therapeutic strategies in patients with STEMI, NSTEMI+CK, and NSTEMI-CK.

## 1. Introduction

The rapid reperfusion of infarct-related artery is critical for patients with acute myocardial infarction (AMI). To avoid delays in the diagnosis and treatment of myocardial infarctions (MI), the European Society of Cardiology (ESC)/the American College of Cardiology (ACC) recommended a new definition of AMI in 2010. This Universal Definition is based on cardiac troponin (cTn) as a biomarker of myocardial injury [1]. However, in Japan, creatine kinase (CK)-based criteria are still widely used in the current clinical setting since ST-elevation myocardial infarctions (STEMI) still constitute the majority of AMI cases [2,3]. A prospective, multicenter, nation-wide registry, the Japanese Registry of Acute Myocardial Infarction Diagnosed by Universal Definition (J-MINUET), was conducted to validate the Universal Definition in the Japanese population [4,5]. Consequently, long-term outcomes were worse in non-STEMI patients compared to those with STEMI. Furthermore, outcomes were not only worse in non-STEMI patients with CK elevation (NSTEMI+CK), but also in non-STEMI patients without CK elevation (NSTEMI-CK) [5]. These results highlight the importance of differentiating between AMI diagnosed by CK-based criteria and AMI diagnosed by cTn-based criteria. Additionally, treatment strategies for secondary prevention could possibly be different among STEMI, NSTEMI+CK, and NSTEMI-CK. Therefore, in the present exploratory study, we conducted a post-hoc analysis of the J-MINUET study to assess predictive factors for long-term outcomes from baseline demographic and clinical characteristics in patients with STEMI, NSTEMI+CK, and NSTEMI-CK.

## 2. Methods

### 2.1. Study Overview and Design

The J-MINUET study enrolled 3283 consecutive patients with AMI from 28 participating Japanese medical institutions between July 2012 and March 2014 [4,5]. Diagnosis of AMI was based on the Third Universal Definition of Myocardial Infarction published in 2012 [6]. Only type 1 AMI (spontaneous MI related to ischemia from primary coronary event) and type 2 AMI (MI secondary to ischemia because of either increased oxygen demand or decreased supply) were included in this registry. In brief, AMI was diagnosed by the rise and/or fall of cardiac biomarkers (preferred: troponin) with at least 1 value above the 99th percentile of the upper reference limit observed together with evidence of myocardial ischemia with at least one of the following: symptoms of ischemia, electrocardiography (ECG) changes indicative of new ischemia, the development of pathological Q waves in the ECG, and imaging evidence of a new loss of viable myocardium or new regional wall motion abnormalities. The type of cTn (cTnT or cTnI) measured depended on the attending physician, and the cut-off value used at each institution was applied. In patients in whom CK was elevated more than twice the upper limit of normal, cTn measurement may not be required. STEMI was diagnosed by the presence of new ST elevations at the J point in at least two contiguous leads ≥2 mm (0.2 mV) in men or ≥1.5 mm (0.15 mV) in women in leads V2–3 and/or ≥1 mm (0.1 mV) in other contiguous chest leads or the limb leads. Furthermore, a new or presumably new left bundle branch block has been considered a STEMI equivalent. Patients without ST-segment elevation, who had elevated CK and/or cTn, were categorized as NSTEMI. Patients with NSTEMI and elevated CK were categorized as NSTEMI+CK, and those without CK elevation but with positive cTn were categorized as NSTEMI-CK. The study protocol was approved by the institutional review board of Dokkyo Medical University (8/1/2013, 24097) and the ethics committees of each participating institution. The study followed the tenets laid out in the Declaration of Helsinki. Informed consent was obtained from all patients. The study was registered in the University Hospital Medical Information Network Clinical Trials registry prior to study commencement (1/3/2013, UMIN000010037).

In the J-MINUET study, 3-year clinical outcomes were evaluated, in which the primary endpoint was the composite of all-cause death, non-fatal MI, non-fatal stroke, heart failure requiring hospitalization, and urgent revascularization for unstable angina [5]. In the present study, we conducted exploratory analyses to determine significant predictors of long-term outcomes in patients with STEMI, NSTEMI+CK, and NSTEMI-CK. These analyses were performed using all of the baseline data for demographic and clinical characteristics evaluated in the J-MINUET study (total of 111 parameters; Supplementary Table S1). Using the Cox proportional hazards model, we extracted significant independent predictors of long-term outcomes and determined regression coefficients for each of these factors. In alignment with previous studies [6], we then proposed a risk score model using regression coefficients for the strict prediction of long-term outcomes in each arm of STEMI, NSTEMI+CK, and NSTEMI-CK.

### 2.2. Statistical Analysis

Exploratory analyses were performed to determine predictors of long-term outcomes in each arm of STEMI, NSTEMI+CK, and NSTEMI-CK. For appropriate analysis of long-term outcomes, we included composite events as demonstrated by the primary endpoint in the J-MINUET study and all-cause death. First, in each arm, the significant determinants of long-term outcomes were extracted by the univariate Cox proportional hazards model, using the incidence of events during 3 years as an objective variable and all of the 111 items for baseline demographic and clinical characteristics as covariates. Using significant covariates extracted by the univariate analyses, we performed multivariate analyses, which were initially conducted without variable selection followed by stepwise variable selection, and calculated hazard ratios (HR) for the incidence of events and 95% confidence intervals (CI). When performing multivariate analysis, if collinearity was present among multiple covariates, we applied one of them to the model. In each of the covariates selected as significant independent predictors for an incidence of events in the multivariate analyses, a regression coefficient was determined. Next, we calculated a risk score using the regression equations as follows: the risk score = Ʃ(categorical or continuous variable × regression coefficient) + Ʃ(dichotomous variable; where yes = 1 or no = 0 × regression coefficient). In each patient, the risk score was calculated for the primary endpoint and all-cause death [7]. Then, median and quartile values were determined in each arm, and patients were classified into two or four subgroups based on the median value or quartile values, respectively. A Kaplan–Meier survival curve was plotted for each subgroup in each arm. Comparisons between two groups or those among four groups were performed using the log-rank test. A *p* value <0.05 was considered statistically significant, and all tests were two-tailed. All statistical analyses were performed using SAS version 9.4 (SAS Institute Inc., Cary, NC, USA).

## 3. Results

Baseline characteristics were measured in each arm of STEMI, NSTEMI+CK, and NSTEMI-CK. In all of 3283 patients enrolled for the J-MINUET study, 2262 patients (68.9%) were categorized as STEMI, 563 patients (17.1%) were categorized as NSTEMI+CK, and 458 patients (14.0%) were categorized as NSTEMI-CK. The major items of baseline characteristics in each arm of STEMI, NSTEMI+CK, and NSTEMI-CK are shown in Supplementary Table S2.

### 3.1. STEMI

**Figure 1:**
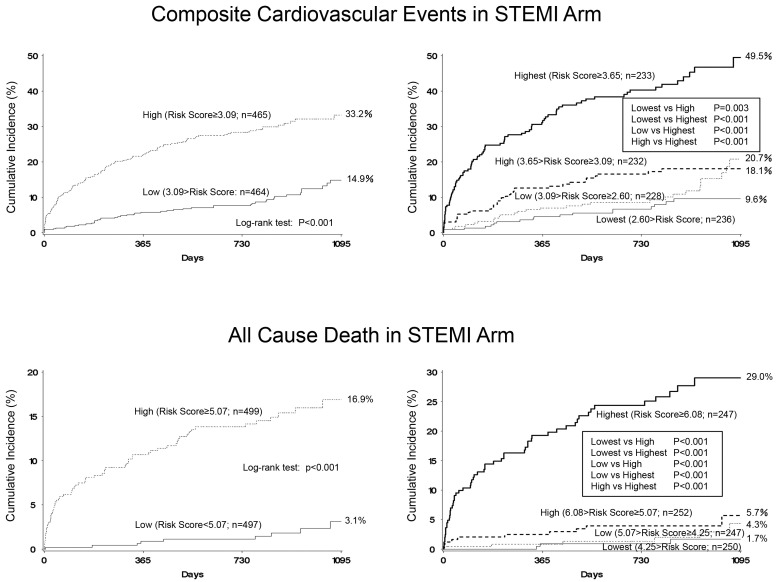
Incidence of composite cardiovascular events and all-cause death in patients with STEMI. Two-group comparison based on the median values of risk scores (high or low) (**left**) and four-group comparison based on the quartile values of risk scores (highest, high, low, or lowest) (**right**).

A multivariate Cox proportional hazards model with stepwise variable selection using variables selected by univariate analysis as covariates showed that older patients, absence of door-to-balloon time < 90 min, higher white blood cell count (WBC) at admission, lower high-density lipoprotein (HDL)-cholesterol level at admission, higher blood glucose level at admission, higher brain natriuretic peptide (BNP) level at any time, and baseline medications of insulin and histamine 2 (H2) blockers at admission were independent predictors of composite cardiovascular events. Additionally, older age, absence of dyslipidemia, absence of door-to-balloon time < 90 min, incidence of acute kidney injury (AKI), higher WBC at admission, higher BNP level at any time, and baseline medication with H2 blockers were predictors of all-cause death (Table 1). Using regression coefficients for each covariate, a risk score was calculated as follows: the risk score for the prediction of composite cardiovascular events = age × 0.04081 + door-to-balloon time < 90 min × (−0.59421) + WBC at admission × 0.0001103 + HDL-cholesterol level at admission × (−0.02155) + blood glucose level at admission × 0.00215 + BNP level at any time × 0.0008597 + baseline insulin medication × 0.75431 + baseline H2 blocker medication × 0.69328; and the risk score for prediction all cause death = age × 0.0646 + dyslipidemia × (−0.9454) + door-to-balloon time < 90 min × (−0.7511) + incidence of acute kidney injury × 1.2870 + WBC at admission × 0.0001 + BNP level at any time × 0.0015 + baseline H2 blocker medication × 1.3757. As a result, the values for the first quartile, median, and third quartile were 2.60, 3.09, and 3.65, respectively, for composite cardiovascular events prediction, and 4.25, 5.07, and 6.08, respectively, for all-cause death prediction. Patients were divided into two subgroups based on the median values. The cumulative incidence of composite cardiovascular events was 33.2% in the group with the high-risk score (risk score ≥ 3.09) and 14.9% in the low group (risk score < 3.09) (log rank test *p* < 0.0001). Furthermore, the cumulative incidence of all-cause death was 16.9% in the high group (risk score ≥ 5.07) and 14.9% in the low group (risk score < 5.07) (log rank test *p* < 0.0001). When patients were divided into 4 subgroups based on the quartile values, the cumulative incidence of composite cardiovascular events was 49.5% in the group with highest quartile values (risk score ≥ 3.65), 18.1% in the high group (3.09 ≤ risk score < 3.65), 20.7% in the low group (2.60 ≤ risk score < 3.09), and 9.6% in the lowest group (<2.60) (log rank test *p* < 0.001). The cumulative incidence of all-cause death was 29.0% in the highest group (risk score ≥ 6.08), 5.7% in the high group (5.07 ≤ risk score < 6.08), 4.3% in the low group (4.25 ≤ risk score < 5.07), and 1.7% in the lowest group (<4.25) (log rank test *p* < 0.001) (Figure 1).

### 3.2. NSTEMI+CK

**Figure 2:**
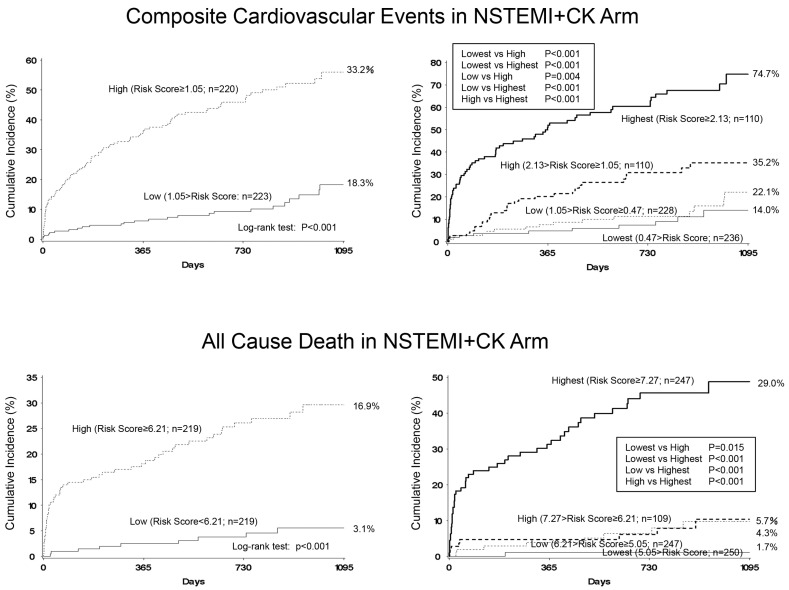
Incidence of composite cardiovascular events and all-cause death in patients with NSTEMI+CK. Two-group comparison based on the median values of risk scores (high or low) (**left**) and four-group comparison based on the quartile values of risk scores (highest, high, low, or lowest) (**right**).

In the multivariate Cox proportional hazards model with stepwise variable selection, higher Killip class, absence of dyslipidemia, history of coronary artery bypass surgery (CABG), history of stroke, higher maximum creatine kinase (max CK) levels, lower low-density lipoprotein (LDL)-cholesterol levels at admission, higher serum potassium (K) at admission, and baseline medications of nitrates were independent predictors of composite cardiovascular events. Additionally, older age, incidence of ventricular tachycardia (VT) or ventricular fibrillation (Vf), higher maximum CK levels, higher uric acid levels at admission, lower LDL-cholesterol levels at admission, and blood glucose levels at admission were predictors of all-cause death (Table 2). Similar to the STEMI group, the risk score was also calculated using the following equation: the risk score for prediction of composite cardiovascular events = Killip class × (I = 0, II = 0.76269, III = 1.37222, IV = 1.92068) + dyslipidemia × (−0.59262) + history of CABG × 0.87018 + history of stroke × 0.71985 + max CK level × 0.0001468 + LDL-cholesterol level at admission × (−0.00994) + K level at admission × 0.47231 + baseline medication of nitrates × 1.18716, and the risk score for prediction all cause death = age × 0.7416 + incidence of VT/Vf × (1.55756) + max CK level × 0.0003 + uric acid level at admission × 0.27987 + LDL-cholesterol level at admission × (−0.01905) + blood glucose level at admission × 0.00524. As a result, the values for the first quartile, median, and third quartile were 0.47, 1.05, and 2.13, respectively, for composite cardiovascular events prediction, and 5.05, 6.21, and 7.27, respectively, for all-cause death prediction. When patients were divided into two subgroups based on the median values, the cumulative incidence of composite cardiovascular events was 56.0% in the group of high risk score (risk score ≥ 1.05) and 18.3% in the low group (risk score < 1.05) (log rank test *p* < 0.0001). Furthermore, the cumulative incidence of all-cause death was 29.6% in the high group (risk score ≥ 6.21) and 5.6% in the low group (risk score < 6.21) (log rank test *p* < 0.0001). When patients were divided into 4 subgroups based on the quartile values, the cumulative incidence of composite cardiovascular events was 49.5% in the group with the highest quartile values (risk score ≥ 2.13), 18.1% in the high group (1.05 ≤ risk score < 2.13), 20.7% in the low group (0.47 ≤ risk score < 1.05), and 9.6% in the lowest group (<0.47) (log rank test *p* < 0.001). Furthermore, the cumulative incidence of all-cause death was 29.0% in the highest group (risk score ≥ 7.27), 5.7% in the high group (6.21 ≤ risk score < 7.27), 4.3% in the low group (5.05 ≤ risk score <6.21), and 1.7% in the lowest group (<5.05) (log rank test *p* < 0.001) (Figure 2).

### 3.3. NSTEMI-CK

**Figure 3:**
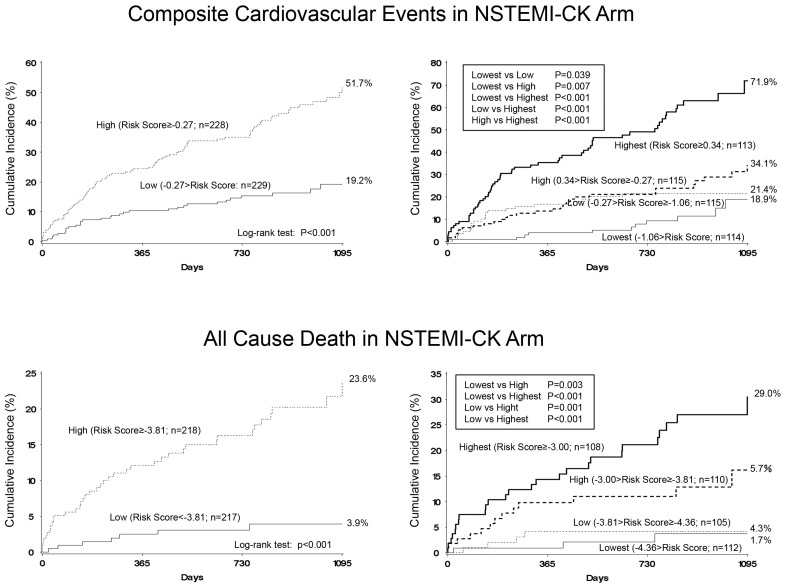
Incidence of composite cardiovascular events and all-cause death in patients with NSTEMI-CK. Two-group comparison based on the median values of risk scores (high or low) (**left**) and four-group comparison based on the quartile values of risk scores (highest, high, low, or lowest) (**right**).

In the multivariate Cox proportional hazards model with stepwise variable selection, the use of intra-aortic balloon pumping (IABP), lower estimated glomerular filtration rate (eGFR) at admission, and baseline medications of antiplatelet drugs and diuretics were independent predictors of composite cardiovascular events. Furthermore, a history of coronary angioplasty, incidence of acute kidney injury, and red blood cell count (RBC) at admission were predictors of all-cause death (Table 3). Next, the risk score was calculated using the following equation: the risk score for prediction of composite cardiovascular events = use of IABP × 0.8788 + eGFR value × (−0.01563) + baseline antiplatelet drugs × 1.04038 + baseline diuretics × 0.84844; for prediction of composite cardiovascular events, and the risk score for prediction of all cause death = history of coronary angioplasty × 1.15805 + incidence of AKI × 1.27068 + RBC at admission × (−0.00969). As a result, the values of the first quartile, median, and third quartile were −1.06, −0.27, and 0.34, respectively, for composite cardiovascular events prediction, and −4.36, −3.81, and −3.00, respectively, for all-cause death prediction. When patients were divided into two subgroups based on the median values, the cumulative incidence of composite cardiovascular events was 51.7% in the group of high risk score (risk score ≥ −0.27) and 19.2% in the low group (risk score < −0.27) (log rank test *p* < 0.0001), and that of all-cause death was 23.6% in the high group (risk score ≥ −3.81) and 3.9% in the low group (risk score < −3.81) (log rank test *p* < 0.0001). When patients were divided into 4 subgroups based on the quartile values, the cumulative incidence of composite cardiovascular events was 71.9% in the group with highest quartile values (risk score ≥ −0.34), 34.1% in the high group (−0.27 ≤ risk score < 0.34), 21.4% in the low group (−1.06 ≤ risk score < −0.27), and 18.9% in the lowest group (< −1.06) (log rank test *p* < 0.001), and that of all-cause death was 30.5% in the highest group (risk score ≥ −3.00), 16.2% in the high group (−3.81 ≤ risk score < −3.00), 4.1% in the low group (−4.36 ≤ risk score < −3.82), and 3.7% in the lowest group (<−4.36) (log rank test *p* < 0.001) (Figure 3).

## 4. Discussion

In this exploratory study, we conducted a post-hoc analysis of the J-MINUET study, using a multivariate Cox proportional hazards model, to determine independent predictive factors for long-term outcomes from a total of 111 baseline demographic and clinical characteristics. Then, we calculated the risk score, a powerful predictor of long-term prognosis, using the regression coefficients for the independent predictive factors. These analyses were performed separately in each arm of STEMI, NSTEMI+CK, and NSTEMI-CK in order to assess the different prognostic factors for long-term outcomes among the various groups. Consequently, we found that prognostic factors were different between patients with STEMI, NSTEMI+CK, and NSTEMI-CK. The prognostic factors also varied between the groups when comparing two objective variables, composite cardiovascular events and all-cause death.

Previous studies have shown that the long-term outcomes of NSTEMI are worse than those of STEMI in Western countries [8,9]. In the Prevention of AtherothrombotiC Incidents Following Ischemic Coronary Attack (PACIFIC) registry, a representative Japanese multicenter registry, the cumulative incidence of cardiovascular events and death from hospital discharge to 1 year or from 1 to 2 years in STEMI patients was similar to that in patients with non-ST elevation acute coronary syndrome, which included cTn-negative unstable angina [3]. The J-MINUET registry, the latest multicenter registry of Japanese patients with acute MI diagnosed by the universal definition, showed that long-term outcomes of NSTEMI were worse than those of STEMI in Japanese patients. Surprisingly, not only were NSTEMI+CK patients associated with worse long-term outcomes compared to STEMI, but so were patients with NSTEMI-CK [5]. Therefore, we hypothesized that clinical and pathophysiological characteristics were different among patients with NSTEMI, NSTEMI+CK, and NSTEMI-CK, and that prognostic factors for long-term outcomes were different among the 3 groups. In the J-MINUET registry, diagnosis of AMI was based on the Third Universal Definition of Acute Myocardial Infarction published in 2012 [6], because the registry was carried out between July 2012 and March 2014. As a current definition, the Fourth Universal Definition of Acute Myocardial Infarction was published in 2018 [10]. However, regarding a concept of cTn-based criteria as demonstrated by the type 1 or type 2 criteria, it is similar to the conventional definitions. Actually, the NSTEMI-CK in the J-MINUET study was applicable to the type 1 or type 2 criteria.

The majority of the independent prognostic factors for long-term outcomes, which were determined by the Cox proportional hazards model analysis, were plausible. However, there were several factors that were incomprehensible. In the STEMI group, baseline medication with H2 blockers at admission was listed as an exacerbation factor for both composite cardiovascular events and all-cause death. Gastric acid suppressive agents such as proton pump inhibitors (PPIs) and H2 blockers are often used in combination with antiplatelet drugs in patients with coronary artery disease. Evidence from previous studies suggests that PPIs might be linked to adverse cardiac events, although a causal relationship is unproven [11,12,13,14]. Conversely, the majority of the literature on H2 blockers indicate favorable effects of medication on cardiovascular outcomes. It has been demonstrated that myocardial histamine H2 receptor activation might promote cardiac fibrosis and apoptosis in preclinical models; thus, H2 blockers may have cardioprotective effects [15,16,17]. H2 blockers have been shown to improve symptoms in patients with heart failure and reduce the incidence of heart failure in persons without cardiovascular disease [17,18]. Additionally, H2 blocker-mediated improvements in anaerobic myocardial metabolism protect against ischemia and reperfusion injury in an animal ischemia/reperfusion model [19]. Therefore, our results that baseline medication with H2 blockers at admission was an exacerbation factor for long-term outcomes are paradoxical. Based on our results, no dyslipidemia and lower LDL-cholesterol levels at admission were exacerbation factors for composite cardiovascular events in the NSTEMI+CK group. Lower LDL-cholesterol levels at admission was also an exacerbation factor for all-cause death. These results also seem paradoxical because higher LDL-cholesterol levels might be associated with adverse cardiovascular events. However, during the acute phase reaction following acute MI, previous studies have reported trends of decreased low-density lipoprotein cholesterol (LDL-C), increased triglycerides, and variable high-density lipoprotein cholesterol (HDL-C) levels. One suggested mechanism explaining the LDL-cholesterol reduction is that changes in liver function including lipoprotein breakdown and excretion may alter LDL-C levels during this inflammatory state [20,21]. Additionally, it has been recently reported that lower LDL cholesterol was associated with in-hospital mortality [22]. Therefore, our data showing that lower LDL-cholesterol levels at admission was an exacerbation factor for long-term outcomes in the NSTEM group might be an acceptable result.

In this exploratory study, we calculated the risk scores by multivariate Cox proportional hazards models to predict long-term prognosis using regression coefficients of the independent covariates selected from baseline demographic and clinical characteristics. Risk scores could effectively and powerfully predict both composite cardiovascular events and all-cause death in each of our groups. We mathematically assessed the risk score, disregarding clinical or pathophysiological mechanisms of association between each covariate and long-term outcomes. The risk score calculations utilizing the methods described above have been established and reported by multiple other studies [7,23,24,25]. Risk score calculations, as conducted in our study, have been previously applied to accurately estimate the onset risk of colorectal cancer in the US Physicians’ Health study [23], assess the risk of diabetic retinopathy in the Indian diabetes cohort by comparison with the conventional Australian diabetes assessment tool [24], and predict in-hospital mortality after cardiac surgery [25]. In the present study, cumulative incidence of composite cardiovascular events and all-cause death were assessed by two-group comparisons based on median values (high or low) and four-group comparisons based on quartile values (highest, high, low, or lowest) in each arm of STEMI, NSTEMI+CK, and NSTEMI-CK. Consequently, the risk score enabled us to achieve effective risk stratification in both two-group and four-group comparisons for the prediction of cardiovascular events and all-cause death in each arm. Risk prediction was most effective in four-group comparisons, as the highest score group showed the greatest risk for cardiovascular events and all-cause death. However, stepwise increases in risk in each group of highest, high, low, and lowest were absent. Overall, risk stratifications for the prediction of long-term outcomes using the risk score as demonstrated in the present study could be promising in the development of therapeutic strategies against STEMI, NSTEMI+CK, and NSTEMI-MI.

### 4.1. Study Limitations

This study has several potential limitations. First, when conducting the Cox proportional hazards models from univariate to multivariate analyses, we included all of the items assessed for baseline demographic and clinical characteristics in the J-MINUET study as covariates. There are many possible confounding factors among those variables. We could not perfectly control these confounding factors, although we applied one of them to the model if the correlation was present among multiple covariates. Second, although the sample size was moderate overall, statistical power may not be sufficient for each of the groups, STEMI, NSTEMI+CK, and NSTEMI-CK, in determining differences in assessment of long-term outcome measurements. Additionally, in the J-MINUET study, the patients categorized as STEMI were 68.9%, the number of which seems higher, compared to that in Western countries, although we cannot explain clearly why the number was higher. Finally, the present study was conducted as an exploratory research study, which disregards clinical or pathophysiological mechanisms of association between each covariate and log-term outcomes. However, these mechanisms should be elucidated when we apply the risk scores we suggested to therapeutic strategies to improve the long-term prognosis for each of STEMI, NSTEMI+CK, and NSTEMI-CK.

### 4.2. Clinical Implications/Conclusions

The J-MINUET study showed that the long-term outcomes of NSTEMI were worse than those of STEMI, not only in patients with NSTEMI+CK, but also in those with NSTEMI-CK [5]. The results suggest that the diagnosis of AMI based on a Universal Definition would be reasonable also in the Japanese population. The present post-hoc analysis demonstrated that prognostic factors were different among patients with STEMI, NSTEMI+CK, and NSTEMI-CK, suggesting that splitting the patients into NSTEMI+CK or NSTEMI-CK groups would be valid. Additionally, we suggested that the risk scores could effectively predict long-term prognosis in each of our groups. The prediction of long-term outcomes, using scored parameters of baseline demographics and clinical characteristics, could be promising. Application of these scores to establish therapeutic strategies could be beneficial in the future.

## Figures and Tables

**Table 1:** Analysis of Cox proportional hazards model with stepwise variable selection for prediction of adverse events in ST elevation acute myocardial infarction (STEMI). BNP: brain natriuretic peptide, HDL: high-density lipoprotein, HR: hazard ratios, WBC: white blood cell count.

Composite Cardiovascular Events		
Variable	HR (95% CI)	*p*
Age; yr	1.042 (1.023–1.060)	<0.001
Door-to-balloon time < 90 min; yes/no	0.552 (0.371–0.821)	0.003
WBC at admission; /mm^3^	1.000 (1.000–1.000)	<0.001
HDL-cholesterol at admission; mg/dL	0.979 (0.961–0.997)	0.023
Blood glucose at admission; mg/dL	1.002 (1.000–1.004)	0.032
BNP at any time; pg/dL	1.001 (1.001–1.001)	<0.001
Insulin; yes/no	2.126 (1.022–4.425)	0.044
H2 blocker; yes/no	2.000 (1.083–3.693)	0.027
All-cause death		
Variable	HR (95% CI)	*p*
Age; yr	1.067 (1.030–1.105)	<0.001
Dyslipidemia; yes/no	0.389 (0.192–0.787)	0.009
Door-to-balloon time < 90 min; yes/no	0.472 (0.238–0.937)	0.032
Acute kidney injury; yes/no	3.622 (1.684–7.790)	<0.001
WBC at admission; /mm^3^	1.000 (1.000–1.000)	0.003
BNP at any time; pg/dL	1.001 (1.001–1.002)	<0.001
H2 blocker; yes/no	3.958 (1.634–9.584)	0.002

**Table 2:** Analysis of Cox proportional hazards model with stepwise variable selection for the prediction of adverse events in non-STEMI with elevated creatine kinase (NSTEMI+CK). VF: ventricular fibrillation, VT: ventricular tachycardia, UA: uric acid, BS: blood sugar.

Composite Cardiovascular Events		
Variable	HR (95% CI)	*p*
Killip class; II/I	2.144 (0.985–4.665)	0.055
Killip class; III/I	3.944 (1.964–7.919)	<0.001
Killip class; IV/I	6.826 (3.377–13.795)	<0.001
Dyslipidemia; yes/no	0.553 (0.342–0.894)	0.016
History of coronary artery bypass surgery; yes/no	2.387 (1.014–5.623)	0.047
	2.054 (1.072–3.935)	0.03
History of stroke; yes/no	1.000 (1.000–1.000)	0.012
maxCK; IU/L	0.990 (0.983–0.998)	<0.001
LDL cholesterol at admission; mg/dL	1.604 (1.077–2.387)	0.02
K at admission; mEq/L	3.278 (1.734–6.195)	<0.001
All cause death		
Variable	HR (95% CI)	*p*
Age; yr	1.077 (1.038–1.118)	<0.001
VT/VF; yes/no	4.747 (1.668–13.512)	0.004
maxCK; IU/L	1.000 (1.000–1.000)	<0.001
UA mg/dL	1.323 (1.092–1.603)	0.004
LDL mg/dL	0.981 (0.969–0.994)	0.003
BS mg/dL	1.005 (1.002–1.008)	<0.001

**Table 3:** Analysis of Cox proportional hazards model with stepwise variable selection for prediction of adverse events in non-STEMI without elevated CK (NSTEMI-CK). eGFR: estimated glomerular filtration rate, RBC: red blood cell count.

Composite Cardiovascular Events		
Variable	HR (95% CI)	*p*
Intra-aortic balloon pumping	2.408 (1.058–5.480)	0.036
eGFR at admission; mL/min/1.73 m^2^	0.984 (0.974–0.995)	0.003
Antiplatelet drugs; yes/no	2.830 (1.631–4.912)	<0.001
Diuretics; yes/no	2.336 (1.281–4.261)	0.006
All-cause death		
Variable	HR (95% CI)	*p*
History of coronary angioplasty; yes/no	3.184 (1.268–7.994)	0.014
Acute kidney injury; yes/no	3.563 (1.049–12.102)	0.042
RBC at admission; yes/no	0.990 (0.983–0.998)	0.009

## Supplementary Materials

The following are available online at https://www.mdpi.com/2077-0383/9/8/2667/s1, Table S1: All of the baseline variables for demographic and clinical characteristics evaluated in the J-MINUET study, Table S2: Baseline characteristics of the study patients enrolled in J-MINUET.
