# Supplementary material for: Prediction of Long-Term Outcomes in ST-Elevation Myocardial Infarction and Non-ST Elevation Myocardial Infarction with and without Creatinine Kinase Elevation—Post-Hoc Analysis of the J-MINUET Study
Source: J Clin Med. 2020 Aug 18;9(8):2667. doi: 10.3390/jcm9082667 (PMC7463547; doi:10.3390/jcm9082667)
Supplement: Supplementary file 1 [file jcm-09-02667-s001.pdf]

Supplementary Materials

Table S1. All of the baseline variables for demographic and clinical characteristics evaluated in the J-MINUET study.

**Age; yr**	**Stent; yes/no**	**Blood glucose; mg/dL**
Gender; men/women	Onset-to -balloon time; min	HbA1c %
Hight; cm	Stent size; mm	Na; mEq/L
Weight; kg	Stent length; mm	K; mEq/L
Onset-to-door time; min	Final TIMI grade; 0/1/2/3	BNP; pg/dL
Systolic blood pressure; mmHg	Final TIMI 3; yes/no	NTpro-BNP; pg/dL
Heart rate; /min	Ventricular tachycardia/ventricular fibrillation at admission; yes/no	Other laboratory findings at any time
Killip class; I/II/III/IV	Atrial fibrillation at admission; yes/no	Fasting blood glucose; mg/dL
Type of myocardial infarction; II/I	Typical symptoms; yes/no	Fasting IRI uU/mL
Past history	Transport; emergency car/transfer/on foot/in-hospital onset	EPA/AA ratio
Hypertension; yes/no	Referral physicians; yes/no	Proteinuria positive; yes/no
Diabetes (HbA1c 6.5); yes/no	Intra-aortic balloon pumping; yes/no	Baseline medications prior to admission
Dyslipidemia; yes/no	Percutaneous cardio-pulmonary support; yes/no	Dual antiplatelet therapy; yes/no
Chronic kidney disease (eGFR<60); yes/no	Acute kidney injury; yes/no	Antiplatelet drugs; yes/no
Hemodialysis; yes/no	cTn measurement at admission; yes/no	Aspirin; yes/no
Smoking habit; yes/no	cTn positive; yes/no	P2Y12 inhibitors; yes/no
Myocardial infarction; yes/no	Tn measurement; yes/no	Anticoagulant drugs; yes/no
Coronary angioplasty; yes/no	Tn positive; yes/no	Direct anticoagulant drugs; yes/no
Coronary artery bypass surgery; yes/no	cTn measurement in hospital; yes/no	Calcium channel blockers
Atrial fibrillation; yes/no	cTn positive in hospital; yes/no	Beta blockers; yes/no
Stroke; yes/no	cTn type; TnI /TnT	Nitrates; yes/no
Ischemic stroke; yes/no	CK> 2 in hospital; yes/no	Nicorandil; yes/no
Peripheral artery disease; yes/no	maxCK; IU/L	Angiotensin converting enzyme inhibitors; yes/no
Atherothrombosis; yes/no	Other laboratory findings at admission	Angiotensin receptor blockers; yes/no
Drug eluting stent; yes/no	WBC; /mm3	Aldosterone antagonists; yes/no
Bare metal stent; yes/no	WBC at admission 10000; yes/no	Diuretics; yes/no
Coronary angioplasty (infarct-related artery); yes/no	RBC; /mm3	Lipid lowering drugs; yes/no
Coronary angioplasty (infarct non-related artery); yes/no	Hemoglobin; mg/dL	Statins; yes/no
Urgent coronary angiography; yes/no	Hematocrit; %	Antidiabetic drugs; yes/no
Affected vessel number; 0/1/2/3	Platelet; /mm3	Insulin; yes/no
Multi-vessel disease; yes/no	BUN; mg/dL	Sulphonylurea drugs; yes/no
Left anterior descending artery as infarct-related artery; yes/no	Creatinine; mg/dL	Pioglitazone; yes/no
Initial TIMI grade; 0/1/2/3	eGFR; mL/min/1.73m2	Metformin; yes/no
Initial TIMI 2 or 3; yes/no	Uric acid; mg/dL	Alpha galactosidase inhibitors; yes/no
Urgent revascularization	Uric acid >7; yes/no	Dipeptidyl peptidase-4 inhibitors; yes/no
Coronary artery bypass surgery; yes/no	Total cholesterol; mg/dL	Hypouricemic drugs; yes/no
Coronary angioplasty; yes/no	Triglyceride; mg/dL	Proton pump inhibitors; yes/no
Door-to-balloon time; min	HDL-cholesterol; mg/dL	H2 blockers; yes/no
Door-to-balloon time<90min; yes/no	LDL-cholesterol; mg/dL	

HbA1c; hemoglobin A1c; TIMI, Thrombolysis In Myocardial Infarction, cTn, cardiac troponin; Tn, troponin; CK, creatine kinase; WBC, white blood cell; RBC, red blood cell; BUN, blood urea nitrogen; eGFR, estimated glomerular filtration rate; HDL, high-density lipoprotein; LDL, low-density lipoprotein; BNP, brain natriuretic peptide; NTpro-BNP, N-terminal pro-brain natriuretic peptide; EPA, eicosapentaenoic acid; AA, arachidonic acid, H2, histamine 2

Table S2. Baseline characteristics of the study patients enrolled in J-MINUET.

	**Total (*n* = 3283)**	**STEMI (*n* = 2262)**	**NSTEMI (*n* = 1021)**	**NSTEMI NSTEMI+CK(*n* = 563)**	**NSTEMI-CK(*n* =458)**
Age (years)	69 (61–78)	68 (60–77)	72 (63–80)	71 (62–80)	73 (63–80)
Male	75.2%	76.8%	71.8%	72.3%	71.2%
Concomitant diseases					
Hypertension	66.5%	63.4%	73.2%	70.3%	76.8%
Diabetes	36.4%	35.0%	39.5%	39.8%	39.1%
Dyslipidemia	51.9%	49.9%	56.4%	53.9%	59.4%
CKD	44.8%	41.9%	51.1%	50.1%	52.4%
Current smoking	34.0%	37.3%	26.9%	26.9%	26.9%
Previous history					
Previous MI	12.1%	9.3%	18.4%	16.0%	21.2%
Previous PCI	15.3%	11.2%	24.4%	21.1%	28.3%
Previous CABG	2.9%	1.8%	5.3%	4.2%	6.6%
Atrial fibrillation	6.0%	5.5%	7.2%	6.4%	8.2%
Stroke	11.3%	10.4%	13.4%	13.5%	13.3%
PAD	4.6%	3.2%	7.8%	7.5%	8.2%
Systolic blood pressure (mmHg)	138 (118–160)	135 (114–157)	144 (124–164)	140 (120–160)	150 (130–170)
Heart rate (beats/min)	77 (65–90)	77 (64–90)	78 (67–92)	80 (67–96)	77 (66–90)
Killip classification					
Class I	75.6%	74.4%	78.3%	71.0%	87.1%
Class II	9.3%	9.5%	8.8%	9.7%	7.7%
Class III	5.4%	5.4%	6.3%	8.8%	3.3%
Class IV	9.7%	11.1%	6.6%	10.4%	2.0%
Time from onset to admission (min)	154 (70–390)	140 (66–334)	195 (79–546)	215 (84–513)	180 (75–557)
Type 2 MI	5.2%	3.0%	10.0%	7.0%	13.6%
Urgent coronary angiography	93.1%	96.9%	84.7%	88.6%	79.9%
Radial approach	32.1%	27.7%	43.0%	32.9%	56.7%
LAD as the infarct artery	44.5%	45.4%	42.4%	38.8%	47.6%
Initial TIMI 0/1 flow	60.4%	70.5%	34.7%	48.9%	14.9%
Multivessel disease	43.7%	41.5%	49.1%	53.1%	43.7%
Primary PCI	85.1%	93.1%	67.4%	72.6%	60.8%
Door-to-balloon time (min)	75 (52–121)	66 (49–92)	137 (88–286)	125 (80–236)	171 (100–399)
Stent use†	90.6%	90.7%	90.5%	89.3%	92.1%
DES use‡	63.2%	59.6%	74.3%	71.2%	78.9%
Final TIMI 3 flow	91.8%	91.0%	94.3%	92.1%	97.4%
Urgent CABG	2.0%	1.1%	4.2%	6.0%	2.0%
Max CK (IU/L)	1447 (518–3,178)	2017 (927–3,848)	505 (181–1,237)	1151 (702–2,076)	161 (101–254)

Data given as % or median (25–75th percentile). CABG, coronary artery bypass graft; CK, creatine kinase; CKD, chronic kidney disease; J MINUET, Japanese Registry of Acute Myocardial Infarction Diagnosed by Universal Definition; LAD, left anterior descending artery; MI, myocardial infarction; NSTEMI, non-STEMI; PAD, peripheral arterial disease; PCI, percutaneous coronary intervention; STEMI, ST-segment elevation myocardial infarction; TIMI, Thrombolysis in Myocardial Infarction.
